# 4-(2,4-Dichlorophenyl)-2-(1*H*-indol-3-yl)-6-methoxypyridine-3,5-dicarbonitrile

**DOI:** 10.1107/S1600536808027670

**Published:** 2008-09-06

**Authors:** P. Ramesh, A. Subbiahpandi, P. Thirumurugan, P. T. Perumal, M. N. Ponnuswamy

**Affiliations:** a Department of Physics, Presidency College (Autonomous), Chennai 600 005, India; b Organic Chemistry Division, Central Leather Research Institute, Adyar, Chennai 600 020, India; c Centre of Advanced Study in Crystallography and Biophysics, University of Madras, Guindy Campus, Chennai 600 025, India

## Abstract

In the title compound, C_22_H_12_Cl_2_N_4_O, the indole ring system and the benzene ring form dihedral angles of 21.18 (7)° and 68.43 (8)°, respectively, with the pyridine ring. The meth­oxy group is coplanar with the pyridine ring. In the crystal structure N—H⋯N inter­molecular hydrogen bonds link the mol­ecules into *C*(10) chains running along [011]. Intramolec­ular C—H⋯N hydrogen bonds are also observed.

## Related literature

For related literature, see: James *et al.* (1991 ▶); Kobayashi *et al.* (1991 ▶); Rajeswaran *et al.* (1999 ▶). For graph-set analysis of hydrogen-bonding patterns, see: Bernstein *et al.* (1995 ▶).
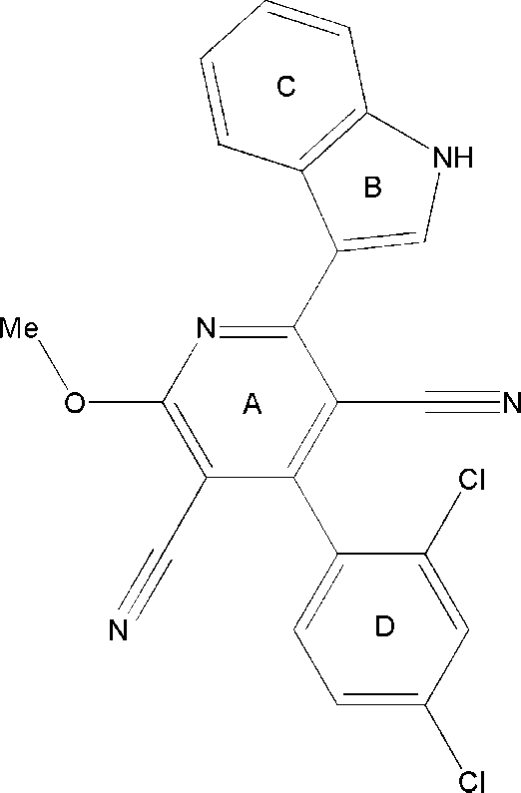


## Experimental

### 

#### Crystal data

C_22_H_12_Cl_2_N_4_O
*M* *_r_* = 419.26
Triclinic, 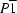

*a* = 9.5394 (2) Å
*b* = 10.0358 (2) Å
*c* = 11.1739 (3) Å
α = 111.994 (1)°
β = 97.303 (1)°
γ = 93.715 (1)°
*V* = 976.46 (4) Å^3^
*Z* = 2
Mo *K*α radiation
μ = 0.35 mm^−1^
*T* = 298 (2) K
0.58 × 0.40 × 0.28 mm

#### Data collection

Bruker APEXII CCD area-detector diffractometer
Absorption correction: multi-scan (*SADABS*; Sheldrick, 2001 ▶) *T*_min_ = 0.821, *T*_max_ = 0.907
10781 measured reflections
3401 independent reflections
3019 reflections with *I* > 2σ(*I*)
*R*_int_ = 0.018

#### Refinement

*R*[*F* ^2^ > 2σ(*F* ^2^)] = 0.033
*wR*(*F* ^2^) = 0.096
*S* = 1.05
3401 reflections
267 parameters
H atoms treated by a mixture of independent and constrained refinement
Δρ_max_ = 0.28 e Å^−3^
Δρ_min_ = −0.37 e Å^−3^

### 

Data collection: *APEX2* (Bruker, 2004 ▶); cell refinement: *APEX2*; data reduction: *SAINT* (Bruker, 2004 ▶); program(s) used to solve structure: *SHELXS97* (Sheldrick, 2008 ▶); program(s) used to refine structure: *SHELXL97* (Sheldrick, 2008 ▶); molecular graphics: *ORTEP-3* (Farrugia, (1997 ▶)); software used to prepare material for publication: *SHELXL97* and *PLATON* (Spek, 2003 ▶).

## Supplementary Material

Crystal structure: contains datablocks global, I. DOI: 10.1107/S1600536808027670/ci2655sup1.cif

Structure factors: contains datablocks I. DOI: 10.1107/S1600536808027670/ci2655Isup2.hkl

Additional supplementary materials:  crystallographic information; 3D view; checkCIF report

## Figures and Tables

**Table 1:** Hydrogen-bond geometry (Å, °)

*D*—H⋯*A*	*D*—H	H⋯*A*	*D*⋯*A*	*D*—H⋯*A*
C9—H9⋯N1	0.93	2.56	3.045 (2)	113
C15—H15⋯N17	0.93	2.56	3.282 (2)	135
N14—H14⋯N25^i^	0.83 (2)	2.22 (2)	2.996 (2)	156 (2)

Symmetry code: (i) 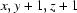.

**Fig. 1.**
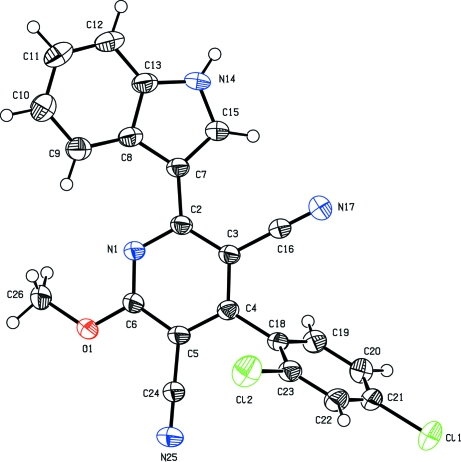
The molecular structure of the title compound. Displacement ellipsoids are drawn at the 50% probability level.

## supplementary crystallographic information

### Comment

Spiro compounds are the naturally occurring substances which exibit many biological properties (Kobayashi *et al.*, 1991; James *et al.*, 1991). Indoles have been proved to display high aldose reductase inhibitory activity (Rajeswaran *et al.*., 1999). In view of this an X-ray diffraction study of the title compound was carrid out.

The indole ring system is essentially planar. The indole ring system and the benzene ring form dihedral angles of 21.18 (7)° and 68.43 (8)°, respectively, with the pyridine ring. The methoxy group is coplanar with the pyridine ring, with the C26—O1—C6—N1 torsion angle being 4.9 (2)°. C—H···N type intramolecular hydrogen bonds are observed in the molecular structure.

In the crystal structure N—H···N intermolecular hydrogen bonds link the molecules into C(10) chains (Bernstein *et al.*, 1995) running along the [0 1 1].

### Experimental

A mixture of 3-cyanoacetyl indole (1 mmol), 2,4 dichlorobenzaldehyde (1 mmol) and sodium hydroxide (1.2 mmol) in methanol was refluxed. After 15 min malanonitrile (1 mmol) was added and the reflux was continued for 4 h. After the completion of the reaction (as monitored by TLC), it was poured into water and extracted with ethyl acetate. The organic layer was dried over sodium sulfate and concentrated under vacuo. The crude product was chromatographed and isolated in 78% yield (90:10, petroleum ether: ethyl acetate). The crude product was recrystallized in ethanol.

### Refinement

The imine H atom was located in a difference map and refined freely. The remaining H atoms were positioned geometrically (C-H = 0.93–0.96 Å) and allowed to ride on their parent atoms, with *U*_iso_(H) = 1.2-1.5*U*_eq_(C).

### Crystal data

**Table:**

C_22_H_12_Cl_2_N_4_O	*Z* = 2
*M**_r_* = 419.26	*F*(000) = 428
Triclinic, *P*1	*D*_x_ = 1.426 Mg m^−^^3^
Hall symbol: -P 1	Mo *K*α radiation, λ = 0.71073 Å
*a* = 9.5394 (2) Å	Cell parameters from 2563 reflections
*b* = 10.0358 (2) Å	θ = 2.2–25.0°
*c* = 11.1739 (3) Å	µ = 0.35 mm^−^^1^
α = 111.994 (1)°	*T* = 298 K
β = 97.303 (1)°	Block, yellow
γ = 93.715 (1)°	0.58 × 0.40 × 0.28 mm
*V* = 976.46 (4) Å^3^	

### Data collection

**Table:**

Bruker APEXII CCD area-detector diffractometer	3401 independent reflections
Radiation source: fine-focus sealed tube	3019 reflections with *I* > 2σ(*I*)
graphite	*R*_int_ = 0.019
ω and φ scans	θ_max_ = 25.0°, θ_min_ = 2.2°
Absorption correction: multi-scan (SADABS; Sheldrick, 2001)	*h* = −11→11
*T*_min_ = 0.821, *T*_max_ = 0.907	*k* = −11→10
10781 measured reflections	*l* = −11→13

### Refinement

**Table:**

Refinement on *F*^2^	Primary atom site location: structure-invariant direct methods
Least-squares matrix: full	Secondary atom site location: difference Fourier map
*R*[*F*^2^ > 2σ(*F*^2^)] = 0.033	Hydrogen site location: inferred from neighbouring sites
*wR*(*F*^2^) = 0.096	H atoms treated by a mixture of independent and constrained refinement
*S* = 1.05	*w* = 1/[σ^2^(*F*_o_^2^) + (0.0487*P*)^2^ + 0.3783*P*] where *P* = (*F*_o_^2^ + 2*F*_c_^2^)/3
3401 reflections	(Δ/σ)_max_ = 0.005
267 parameters	Δρ_max_ = 0.29 e Å^−^^3^
0 restraints	Δρ_min_ = −0.37 e Å^−^^3^

### Special details

**Table:**

Geometry. All e.s.d.'s (except the e.s.d. in the dihedral angle between two l.s. planes) are estimated using the full covariance matrix. The cell e.s.d.'s are taken into account individually in the estimation of e.s.d.'s in distances, angles and torsion angles; correlations between e.s.d.'s in cell parameters are only used when they are defined by crystal symmetry. An approximate (isotropic) treatment of cell e.s.d.'s is used for estimating e.s.d.'s involving l.s. planes.
Refinement. Refinement of *F*^2^ against ALL reflections. The weighted *R*-factor *wR* and goodness of fit *S* are based on *F*^2^, conventional *R*-factors *R* are based on *F*, with *F* set to zero for negative *F*^2^. The threshold expression of *F*^2^ > σ(*F*^2^) is used only for calculating *R*-factors(gt) *etc*. and is not relevant to the choice of reflections for refinement. *R*-factors based on *F*^2^ are statistically about twice as large as those based on *F*, and *R*- factors based on ALL data will be even larger.

### Fractional atomic coordinates and isotropic or equivalent isotropic displacement parameters (Å^2^)

**Table:**

	*x*	*y*	*z*	*U*_iso_*/*U*_eq_	
Cl1	−0.20213 (5)	0.05813 (6)	0.06721 (6)	0.06710 (19)	
Cl2	0.18710 (5)	0.43669 (6)	0.01921 (5)	0.05569 (16)	
O1	0.71041 (12)	0.47517 (13)	0.22771 (11)	0.0440 (3)	
N1	0.60609 (13)	0.63393 (14)	0.38834 (12)	0.0322 (3)	
C2	0.49048 (16)	0.66990 (16)	0.44682 (14)	0.0294 (3)	
C3	0.36036 (15)	0.57682 (16)	0.39683 (14)	0.0293 (3)	
C4	0.34811 (16)	0.45252 (16)	0.28224 (14)	0.0294 (3)	
C5	0.46814 (16)	0.42069 (17)	0.22319 (15)	0.0332 (4)	
C6	0.59570 (16)	0.51473 (17)	0.28335 (15)	0.0327 (3)	
C7	0.51214 (16)	0.80627 (17)	0.55884 (15)	0.0322 (3)	
C8	0.62593 (17)	0.92294 (16)	0.58897 (15)	0.0332 (3)	
C9	0.73824 (19)	0.94826 (18)	0.52844 (17)	0.0417 (4)	
H9	0.7519	0.8801	0.4486	0.050*	
C10	0.8286 (2)	1.0759 (2)	0.5888 (2)	0.0505 (5)	
H10	0.9042	1.0927	0.5493	0.061*	
C11	0.8089 (2)	1.18028 (19)	0.7078 (2)	0.0516 (5)	
H11	0.8719	1.2651	0.7464	0.062*	
C12	0.6985 (2)	1.16004 (19)	0.76875 (18)	0.0474 (4)	
H12	0.6843	1.2300	0.8474	0.057*	
C13	0.60840 (18)	1.03058 (18)	0.70822 (16)	0.0380 (4)	
N14	0.49146 (17)	0.98300 (16)	0.74801 (15)	0.0456 (4)	
H14	0.462 (2)	1.023 (2)	0.818 (2)	0.057 (6)*	
C15	0.43425 (18)	0.85081 (18)	0.66041 (16)	0.0399 (4)	
H15	0.3544	0.7974	0.6671	0.048*	
C16	0.23671 (17)	0.61040 (17)	0.45772 (15)	0.0349 (4)	
N17	0.13813 (16)	0.63647 (17)	0.50645 (16)	0.0519 (4)	
C18	0.21094 (15)	0.35534 (16)	0.22670 (14)	0.0302 (3)	
C19	0.15904 (18)	0.27834 (18)	0.29551 (16)	0.0380 (4)	
H19	0.2100	0.2892	0.3759	0.046*	
C20	0.03299 (19)	0.18576 (19)	0.24684 (18)	0.0441 (4)	
H20	0.0002	0.1335	0.2932	0.053*	
C21	−0.04298 (17)	0.17221 (18)	0.12898 (18)	0.0427 (4)	
C22	0.00404 (17)	0.24803 (19)	0.05835 (17)	0.0425 (4)	
H22	−0.0485	0.2383	−0.0211	0.051*	
C23	0.13088 (17)	0.33881 (17)	0.10799 (15)	0.0353 (4)	
C24	0.46601 (17)	0.29503 (19)	0.10682 (16)	0.0404 (4)	
N25	0.46376 (18)	0.19450 (19)	0.01436 (16)	0.0597 (5)	
C26	0.84527 (18)	0.5613 (2)	0.29323 (18)	0.0490 (5)	
H26A	0.8435	0.6564	0.2920	0.073*	
H26B	0.9201	0.5162	0.2491	0.073*	
H26C	0.8619	0.5687	0.3822	0.073*	

### Atomic displacement parameters (Å^2^)

**Table:**

	*U*^11^	*U*^22^	*U*^33^	*U*^12^	*U*^13^	*U*^23^
Cl1	0.0368 (3)	0.0635 (3)	0.0759 (4)	−0.0164 (2)	0.0051 (2)	0.0037 (3)
Cl2	0.0581 (3)	0.0665 (3)	0.0449 (3)	−0.0046 (2)	−0.0027 (2)	0.0297 (2)
O1	0.0292 (6)	0.0475 (7)	0.0372 (6)	0.0012 (5)	0.0101 (5)	−0.0049 (5)
N1	0.0281 (7)	0.0327 (7)	0.0266 (7)	0.0013 (5)	0.0038 (5)	0.0017 (5)
C2	0.0310 (8)	0.0295 (7)	0.0237 (7)	0.0045 (6)	0.0040 (6)	0.0058 (6)
C3	0.0292 (8)	0.0296 (8)	0.0248 (7)	0.0043 (6)	0.0047 (6)	0.0053 (6)
C4	0.0286 (8)	0.0301 (8)	0.0251 (7)	0.0030 (6)	0.0010 (6)	0.0069 (6)
C5	0.0318 (8)	0.0322 (8)	0.0261 (8)	0.0027 (6)	0.0044 (6)	0.0008 (6)
C6	0.0292 (8)	0.0354 (8)	0.0272 (8)	0.0033 (6)	0.0059 (6)	0.0049 (6)
C7	0.0315 (8)	0.0308 (8)	0.0264 (8)	0.0031 (6)	0.0025 (6)	0.0031 (6)
C8	0.0358 (8)	0.0287 (8)	0.0286 (8)	0.0041 (6)	0.0000 (6)	0.0056 (6)
C9	0.0469 (10)	0.0356 (9)	0.0391 (9)	0.0028 (7)	0.0097 (8)	0.0099 (7)
C10	0.0497 (11)	0.0440 (10)	0.0567 (12)	−0.0032 (8)	0.0108 (9)	0.0190 (9)
C11	0.0547 (11)	0.0342 (9)	0.0547 (12)	−0.0084 (8)	−0.0026 (9)	0.0104 (8)
C12	0.0556 (11)	0.0327 (9)	0.0384 (10)	−0.0009 (8)	−0.0011 (8)	0.0002 (7)
C13	0.0412 (9)	0.0329 (8)	0.0309 (8)	0.0031 (7)	0.0012 (7)	0.0040 (7)
N14	0.0491 (9)	0.0395 (8)	0.0308 (8)	0.0010 (7)	0.0124 (7)	−0.0071 (6)
C15	0.0384 (9)	0.0378 (9)	0.0308 (8)	−0.0009 (7)	0.0071 (7)	−0.0006 (7)
C16	0.0324 (8)	0.0324 (8)	0.0305 (8)	−0.0003 (6)	0.0045 (7)	0.0027 (6)
N17	0.0397 (8)	0.0519 (9)	0.0508 (9)	0.0006 (7)	0.0172 (7)	0.0026 (7)
C18	0.0278 (8)	0.0282 (7)	0.0275 (8)	0.0042 (6)	0.0051 (6)	0.0026 (6)
C19	0.0364 (9)	0.0393 (9)	0.0333 (9)	0.0022 (7)	0.0036 (7)	0.0097 (7)
C20	0.0415 (9)	0.0404 (9)	0.0474 (10)	−0.0012 (7)	0.0133 (8)	0.0126 (8)
C21	0.0287 (8)	0.0380 (9)	0.0464 (10)	−0.0009 (7)	0.0060 (7)	0.0001 (7)
C22	0.0326 (9)	0.0467 (10)	0.0352 (9)	0.0020 (7)	−0.0033 (7)	0.0045 (8)
C23	0.0339 (8)	0.0361 (8)	0.0297 (8)	0.0039 (6)	0.0029 (6)	0.0067 (7)
C24	0.0306 (8)	0.0408 (9)	0.0350 (9)	−0.0004 (7)	0.0067 (7)	−0.0017 (8)
N25	0.0475 (9)	0.0546 (10)	0.0464 (10)	−0.0016 (7)	0.0124 (7)	−0.0152 (8)
C26	0.0291 (9)	0.0586 (11)	0.0441 (10)	−0.0027 (8)	0.0088 (7)	0.0032 (8)

### Geometric parameters (Å, °)

**Table:**

Cl1—C21	1.7345 (16)	C11—C12	1.369 (3)
Cl2—C23	1.7418 (17)	C11—H11	0.93
O1—C6	1.3363 (19)	C12—C13	1.390 (2)
O1—C26	1.443 (2)	C12—H12	0.93
N1—C6	1.3119 (19)	C13—N14	1.373 (2)
N1—C2	1.3533 (19)	N14—C15	1.347 (2)
C2—C3	1.417 (2)	N14—H14	0.83 (2)
C2—C7	1.448 (2)	C15—H15	0.93
C3—C4	1.399 (2)	C16—N17	1.142 (2)
C3—C16	1.432 (2)	C18—C19	1.388 (2)
C4—C5	1.389 (2)	C18—C23	1.390 (2)
C4—C18	1.491 (2)	C19—C20	1.384 (2)
C5—C6	1.411 (2)	C19—H19	0.93
C5—C24	1.429 (2)	C20—C21	1.374 (3)
C7—C15	1.385 (2)	C20—H20	0.93
C7—C8	1.452 (2)	C21—C22	1.378 (3)
C8—C9	1.396 (2)	C22—C23	1.382 (2)
C8—C13	1.404 (2)	C22—H22	0.93
C9—C10	1.378 (2)	C24—N25	1.139 (2)
C9—H9	0.93	C26—H26A	0.96
C10—C11	1.395 (3)	C26—H26B	0.96
C10—H10	0.93	C26—H26C	0.96
			
C6—O1—C26	117.54 (12)	N14—C13—C12	129.16 (16)
C6—N1—C2	119.61 (13)	N14—C13—C8	107.90 (14)
N1—C2—C3	119.97 (13)	C12—C13—C8	122.94 (16)
N1—C2—C7	115.10 (13)	C15—N14—C13	109.99 (14)
C3—C2—C7	124.94 (13)	C15—N14—H14	122.7 (15)
C4—C3—C2	120.27 (13)	C13—N14—H14	127.0 (15)
C4—C3—C16	118.28 (13)	N14—C15—C7	109.82 (15)
C2—C3—C16	121.39 (13)	N14—C15—H15	125.1
C5—C4—C3	118.09 (14)	C7—C15—H15	125.1
C5—C4—C18	120.99 (13)	N17—C16—C3	179.60 (19)
C3—C4—C18	120.91 (13)	C19—C18—C23	117.88 (14)
C4—C5—C6	118.03 (13)	C19—C18—C4	119.46 (14)
C4—C5—C24	121.96 (14)	C23—C18—C4	122.65 (14)
C6—C5—C24	119.99 (14)	C20—C19—C18	121.33 (16)
N1—C6—O1	120.15 (13)	C20—C19—H19	119.3
N1—C6—C5	123.88 (14)	C18—C19—H19	119.3
O1—C6—C5	115.97 (13)	C21—C20—C19	119.01 (17)
C15—C7—C2	128.05 (15)	C21—C20—H20	120.5
C15—C7—C8	106.02 (13)	C19—C20—H20	120.5
C2—C7—C8	125.90 (14)	C20—C21—C22	121.45 (15)
C9—C8—C13	118.23 (15)	C20—C21—Cl1	119.68 (15)
C9—C8—C7	135.50 (14)	C22—C21—Cl1	118.87 (14)
C13—C8—C7	106.27 (14)	C21—C22—C23	118.65 (16)
C10—C9—C8	118.98 (16)	C21—C22—H22	120.7
C10—C9—H9	120.5	C23—C22—H22	120.7
C8—C9—H9	120.5	C22—C23—C18	121.66 (16)
C9—C10—C11	121.41 (18)	C22—C23—Cl2	118.11 (13)
C9—C10—H10	119.3	C18—C23—Cl2	120.21 (12)
C11—C10—H10	119.3	N25—C24—C5	179.6 (2)
C12—C11—C10	121.15 (17)	O1—C26—H26A	109.5
C12—C11—H11	119.4	O1—C26—H26B	109.5
C10—C11—H11	119.4	H26A—C26—H26B	109.5
C11—C12—C13	117.28 (16)	O1—C26—H26C	109.5
C11—C12—H12	121.4	H26A—C26—H26C	109.5
C13—C12—H12	121.4	H26B—C26—H26C	109.5
			
C6—N1—C2—C3	1.9 (2)	C8—C9—C10—C11	−0.8 (3)
C6—N1—C2—C7	−177.70 (14)	C9—C10—C11—C12	−0.3 (3)
N1—C2—C3—C4	−4.2 (2)	C10—C11—C12—C13	0.9 (3)
C7—C2—C3—C4	175.33 (14)	C11—C12—C13—N14	179.70 (19)
N1—C2—C3—C16	178.90 (14)	C11—C12—C13—C8	−0.5 (3)
C7—C2—C3—C16	−1.6 (2)	C9—C8—C13—N14	179.26 (15)
C2—C3—C4—C5	2.7 (2)	C7—C8—C13—N14	−0.18 (19)
C16—C3—C4—C5	179.73 (15)	C9—C8—C13—C12	−0.6 (3)
C2—C3—C4—C18	−178.57 (14)	C7—C8—C13—C12	179.97 (16)
C16—C3—C4—C18	−1.6 (2)	C12—C13—N14—C15	179.98 (19)
C3—C4—C5—C6	0.8 (2)	C8—C13—N14—C15	0.1 (2)
C18—C4—C5—C6	−177.91 (14)	C13—N14—C15—C7	0.0 (2)
C3—C4—C5—C24	179.19 (15)	C2—C7—C15—N14	177.82 (16)
C18—C4—C5—C24	0.5 (2)	C8—C7—C15—N14	−0.1 (2)
C2—N1—C6—O1	−177.39 (14)	C5—C4—C18—C19	112.23 (18)
C2—N1—C6—C5	1.9 (2)	C3—C4—C18—C19	−66.4 (2)
C26—O1—C6—N1	4.9 (2)	C5—C4—C18—C23	−68.3 (2)
C26—O1—C6—C5	−174.42 (16)	C3—C4—C18—C23	113.05 (17)
C4—C5—C6—N1	−3.3 (3)	C23—C18—C19—C20	1.2 (2)
C24—C5—C6—N1	178.32 (16)	C4—C18—C19—C20	−179.29 (15)
C4—C5—C6—O1	176.04 (15)	C18—C19—C20—C21	−1.1 (3)
C24—C5—C6—O1	−2.4 (2)	C19—C20—C21—C22	0.3 (3)
N1—C2—C7—C15	−158.09 (17)	C19—C20—C21—Cl1	−179.34 (13)
C3—C2—C7—C15	22.4 (3)	C20—C21—C22—C23	0.3 (3)
N1—C2—C7—C8	19.4 (2)	Cl1—C21—C22—C23	179.99 (12)
C3—C2—C7—C8	−160.10 (15)	C21—C22—C23—C18	−0.2 (2)
C15—C7—C8—C9	−179.15 (19)	C21—C22—C23—Cl2	−178.73 (13)
C2—C7—C8—C9	2.9 (3)	C19—C18—C23—C22	−0.5 (2)
C15—C7—C8—C13	0.16 (18)	C4—C18—C23—C22	179.96 (14)
C2—C7—C8—C13	−177.80 (15)	C19—C18—C23—Cl2	177.97 (12)
C13—C8—C9—C10	1.2 (3)	C4—C18—C23—Cl2	−1.5 (2)
C7—C8—C9—C10	−179.58 (18)		

### Hydrogen-bond geometry (Å, °)

**Table:**

*D*—H···*A*	*D*—H	H···*A*	*D*···*A*	*D*—H···*A*
C9—H9···N1	0.93	2.56	3.045 (2)	113
C15—H15···N17	0.93	2.56	3.282 (2)	135
N14—H14···N25^i^	0.83 (2)	2.22 (2)	2.996 (2)	156 (2)

Symmetry codes:  (i) *x*, *y*+1, *z*+1.

## References

[bb1] Bernstein, J., Davis, R. E., Shimoni, L. & Chang, N. L. (1995). *Angew. Chem. Int. Ed. Engl.* **34**, 1555–1573.

[bb2] Bruker (2004). *APEX2* and *SAINT* Bruker AXS Inc., Madison, Wisconsin, USA.

[bb3] Farrugia, L. J. (1997). *J. Appl. Cryst.* **30**, 565.

[bb4] James, D., Kunz, H. B. & Faulkner, D. (1991). *J. Nat. Prod.* **54**, 1137–1140. 10.1021/np50076a040 1791478

[bb5] Kobayashi, J., Tsuda, M., Agmi, K., Shigmori, H., Ishibashi, M., Sasaki, T. & Mikami, Y. (1991). *Tetrahedron*, **47**, 6617–6622.

[bb6] Rajeswaran, W. G., Labroo, R. B., Cohen, L. A. & King, M. M. (1999). *J. Org. Chem.* **64**, 1369–1371.

[bb7] Sheldrick, G. M. (2001). *SADABS* University of Göttingen, Germany.

[bb8] Sheldrick, G. M. (2008). *Acta Cryst.* A**64**, 112–122. 10.1107/S0108767307043930 18156677

[bb9] Spek, A. L. (2003). *J. Appl. Cryst.* **36**, 7–13.

